# Supplementary material for: Causal Relationship between Aging and Anorexia Nervosa: A White-Matter-Microstructure-Mediated Mendelian Randomization Analysis
Source: Biomedicines. 2024 Aug 16;12(8):1874. doi: 10.3390/biomedicines12081874 (PMC11351342; doi:10.3390/biomedicines12081874)
Supplement: Supplementary file 1 [file biomedicines-12-01874-s001.zip › Supplementary Figure/Figure S8/Leaveoneout plot of MR analysis for TL to ubm-b-1503.pdf]

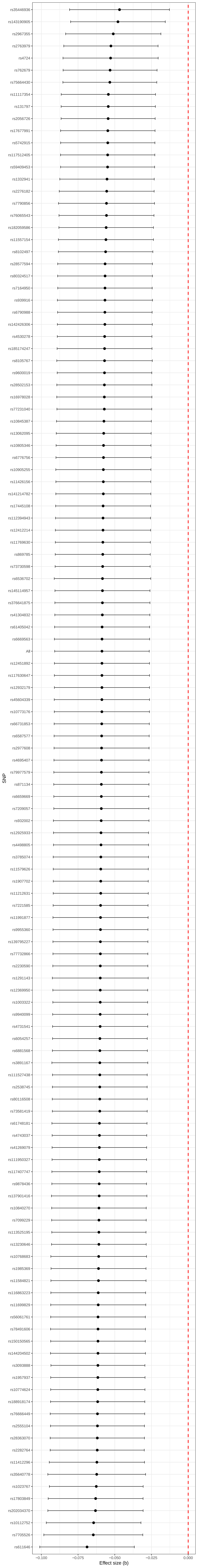
rs35446936
rs143190905
rs2967355
rs2763979
rs4724
rs762679
rs75664430
rs11117354
rs131797
rs2056726
rs17677991
rs5742915
rs117512405
rs59409453
rs1332941
rs2276182
rs7790856
rs76065543
rs182059586
rs11557154
rs8102497
rs28577594
rs80324517
rs7164950
rs939916
rs6790988
rs142426306
rs4530278
rs185174247
rs8105767
rs9600019
rs28502153
rs16978028
rs77231040
rs10845387
rs13062095
rs10805346
rs6776756
rs10905255
rs11426156
rs141214782
rs17445108
rs112394943
rs12412214
rs11769630
rs869785
rs73730598
rs6536702
rs145114957
rs376641875
rs41304832
rs61405042
rs6669563
All
rs12451892
rs117630647
rs12932179
rs45604339
rs10773176
rs66731853
rs6587577
rs2977608
rs4695407
rs79977579
rs871134
rs6659669
rs7209057
rs932002
rs12925933
rs4498805
rs3785074
rs11579626
rs1907702
rs11212631
rs7221585
rs11991877
rs9955360
rs139795227
rs77732866
rs2230590
rs1291143
rs12369950
rs1003322
rs9940099
rs4731541
rs6054257
rs6881568
rs3891167
rs111527438
rs2538745
rs80116508
rs73581419
rs61748181
rs4743037
rs41269079
rs111950327
rs117407747
rs9878436
rs137901416
rs10840270
rs7099229
rs113525195
rs13230646
rs10768683
rs1985369
rs11584821
rs116863223
rs11699829
rs56061761
rs78491606
rs150150565
rs144204502
rs3093888
rs1957937
rs10774624
rs188918174
rs76666449
rs2555104
rs28363070
rs2282764
rs11412296
rs35640778
rs1023767
rs17803849
rs202034370
rs10112752
rs7705526
rs611646
SNP
-0.100
-0.075
-0.050
-0.025
0.000
Effect size (b)
